# Zuranolone for postpartum depression: a systematic review and meta-analysis of two randomized studies

**DOI:** 10.61622/rbgo/2024rbgo79

**Published:** 2024-12-04

**Authors:** Juliana Almeida Oliveira, Karine Eskandar, Marcos Aurélio Araújo Freitas, Chris Elizabeth Philip

**Affiliations:** 1 Universidade Federal de Minas Gerais Belo Horizonte MG Brasil Universidade Federal de Minas Gerais, Belo Horizonte, MG, Brasil.; 2 Pontifícia Universidade Católica do Paraná Curitiba PR Brasil Pontifícia Universidade Católica do Paraná, Curitiba, PR, Brasil.; 3 Universidade Estadual da Região Tocantina do Maranhão Imperatriz MA Brasil Universidade Estadual da Região Tocantina do Maranhão, Imperatriz, MA, Brasil.; 4 Beaumont Hospital Department of Gynaecology Dublin Ireland Department of Gynaecology, Beaumont Hospital, Beaumont Rd, Dublin, Ireland.

**Keywords:** Major depressive disorder, Postpartum depression, Synthetic neurosteroid, Zuranolone

## Abstract

**Objective:**

To evaluate the maternal outcomes in women with postpartum depression using zuranolone, the first oral medication indicated to treat postpartum depression.

**Methods:**

We conducted a systematic search in September 2023, on Pubmed, Embase and Cochrane Trials. We included randomized controlled trials comparing the effectiveness and safety of zuranolone versus placebo in women with postpartum depression. No time or language restrictions were applied. 297 results were retrieved, of which 11 papers were selected and fully reviewed by two authors. Review Manager 5 was used for statistical analysis and Cochrane Risk-of-bias tool for randomized trials was applied for quality assessment.

**Results:**

We included 2 studies, with 346 women, of whom 174 (50.2%) were treated with zuranolone. Zuranolone was significantly associated to an improvement of Clinical Global Impression response rate; Hamilton Depression Rating Scale 15 days and 45-day remission, 3-day, 15-day, and 45-day symptom remission, and reduction in the dose of antidepressants. As for safety outcomes, it was noticed that zuranolone increases sedation risk, which can be dose related. No significant differences were found for other adverse events.

**Conclusion:**

These findings suggest that zuranolone might present a safe and effective medication for out-of-hospital treatment of PPD. Sedation effects need to be further assessed.

## Introduction

Postpartum depression (PPD) affects 13% to 19% of women who have recently given birth.^(1)^ Diagnosis of PPD is typically made when a woman exhibits symptoms such as a persistent low mood, feelings of sadness, despair, and a sense of worthlessness, often linked to childbirth and occur within the first four weeks after giving birth.^(2)^ Additionally, major depressive disorder (MDD) is linked to reduced quality of life and compromised social functioning. This can affect aspects of daily living, including sexual health and adherence to treatment plans.^(3)^

Although PPD arises from multiple factors, current medications primarily target biological aspects.^(4)^ The exact pathophysiology of PPD remains elusive; however, prevailing theories suggest that a rapid decline in pregnancy hormones like progesterone, estradiol, and cortisol may be a key biological trigger.^(5)^ Additionally, other hypotheses focus on neuroendocrine alterations that impair gamma-aminobutyric acid (GABA) signaling and result in reduced levels of allopregnanolone.^(5)^

Recently, the United States Food and Drug Administration (FDA) approved Zuranolone as the first oral medication indicated to treat postpartum depression in adults.^(6)^ Zuranolone belongs to a class known as neuroactive steroid GABA receptor modulators. It increases the expression of synaptic and extrasynaptic GABA-A receptors whose reduced function is related to depression. This improvement in inhibitory GABAergic signaling contributes to its therapeutic effect. Furthermore, Zuranolone's pharmacokinetic profile enables a once-a-day use.^(7)^ Based on this, the American College of Obstetrics and Gynecology (ACOG) recommends zuranolone for managing depression in the postpartum period, specifically targeting cases that begin in the third trimester or within four weeks following childbirth.^(6)^

Given the need for better assessment of the zuranolone effect in PPD, we performed a systematic review and meta-analysis comparing the effectiveness and safety of zuranolone versus placebo in women with postpartum depression.

## Methods

We performed a systematic review and meta-analysis according to recommendations from the Cochrane Collaboration and the Preferred Reporting Items for Systematic Reviews and Meta-Analysis (PRISMA). The protocol was prospectively registered in the International Prospective Register of Systematic Reviews.

We systematically searched PubMed, Embase, and Cochrane Central Register of Controlled trials in September 2023, using the medical subject heading term "zuranolone", and its similar terms. In May 2024 we manually search for any novel studies on the topic, but none were found. The complete search strategy is reported in chart S1. We also utilized the "backward snowballing" technique, searching for additional eligible studies by reviewing references from prior publications. We included studies that met all the following eligibility criteria: (1) study population composed of pregnant women; (2) head-to-head comparison of zuranolone versus placebo; and (3) clinical studies. There was no time or language restriction. We excluded studies with overlapping patient populations. Study screening was carried out independently by two authors, following the predefined search criteria. Eventual conflicts were resolved by consensus among the authors.

Two authors extracted outcomes and baseline characteristics data independently and ensured that the data was consistent for statistical analysis. Patient-level data was not requested. The endpoints of interest were: (1) Clinical Global Impression (CGI) response rate; (2) Hamilton Depression Rating Scale (HAM-D) remission after 15 and 45 days; (3) remission of PPD after 3 days, 15 days, and 45 days; and (7) dose reduction. Safety outcomes included: (1) diarrhea; (2) dizziness; (3) headache; (4) nausea; (5) sedation; (6) somnolence; and (7) upper respiratory tract infection. There were no planned subgroup analyses.

The applied HAM-D scale had 17 variables (HAMD-17) that can be found in different intensities, that vary in two different scores grades, depending on the variable: (1) absent, mild or trivial, moderate, or severe; and (2) absent, slight or doubtful, or clearly present.^(8)^ A score of 26 or higher was required for diagnosis. Remission was defined as a reduction of ≥ 7 points in the HAMD-D scale from the baseline score. We also examined the delta in HAMD-17 score from baseline to 15- and 45-days as a continuous outcome.^(8)^

The CGI is a stand-alone assessment created to evaluate, in studies, the patient's global functioning before and after the start of the intervention. It considers the patient's history and behavior, psychosocial circumstances, and symptoms and how they influence the patient's ability to function. Improvement in the CGI scale was defined as a response of 1 (very much improved) or 2 (much improved) since onset of treatment.^(9)^

Der Simonian and Laird random effects model was employed to compute pooled outcomes for binary endpoints, using odds ratios (OR) and 95% confidence intervals (CI) as effect size measures. Statistical heterogeneity was evaluated through I2 and Cochran Q test, deeming heterogeneity significant if the p-value was < 0.10 and I^2^ exceeded 25%. Sensitivity analyses were conducted employing the leave-one-out strategy and Baujat plots. Statistical analysis was performed using Review Manager 5.1 (Nordic Cochrane Centre, The Cochrane Collaboration, Copenhagen, Denmark) and RStudio (PBC, Boston, MA).

The quality of studies included was appraised using the Cochrane Risk-of-bias tool for randomized trials (RoB 2). Two authors completed the risk assessment independently, and disagreements were resolved by discussing the discrepancies. Small study effect (publication bias) could not be assessed due to the small number of studies.

## Results

**Figure 1:**
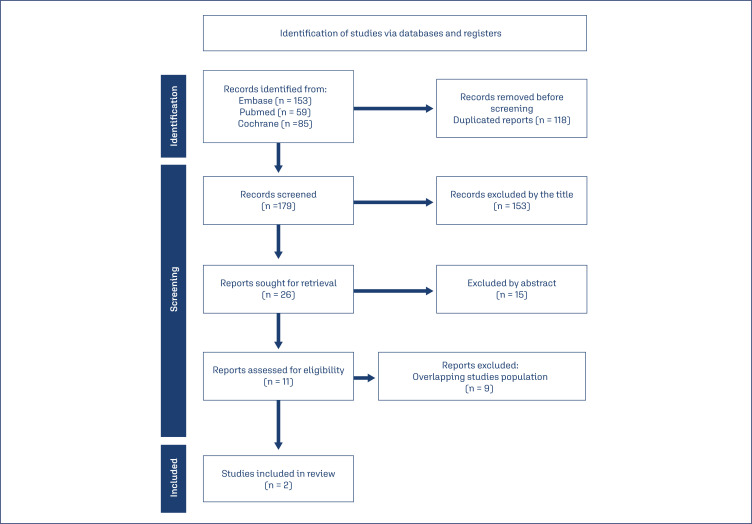
Flow diagram of the screening process

The search strategy yielded 297 results, of which 11 papers were selected after title and abstract screening and fully reviewed according to the inclusion criteria (Figure 1). We included 2 randomized controlled trials, with 346 patients, of whom 174 (50.2%) used zuranolone and 172 with placebo. Both studies included similar populations: women aged 18 to 45 years, with major depressive onset during the third trimester of pregnancy, or ≤4 weeks after delivery. The 2021 study used a 30mg dose while the 2023 one used 50mg. Additional baseline characteristics can be better seen in table 1.

**Table 1:** Baseline characteristics of the included studies

Study	N of patients (Zuranolone*/* control)	BMI* (kg/m^2^)	Age* (years)	Baseline use of antidepressants, n (Zuranolone/ control)	PPD in third trimester, n (Zuranolone/ control)	PPD within 4 weeks after delivery, n (Zuranolone/ control)	Race, n (Zuranolone/control)		
							White	Black	Other
Deligiannidis et al. (2021^)(4^)	76/74	30.7 ± 7.05	28.36 ± 5.42	16/13	32/31	44/43	44/40	31/31	1/3
Deligiannidis et al. (2023^)(10^)	98/98	30.25 ± 6.32	30.5 ± 5.96	15/15	34/31	64/67	68/69	25/18	5/11

* Mean and Standard deviation; Other data represented with number (percentage). Body Mass Index (BMI); Postpartum depression (DPP)

### Efficacy outcomes

**Figure 2:**
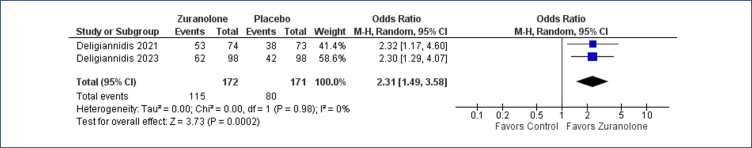
The CGI response rate was higher in the zuranolone group

**Figure 3:**
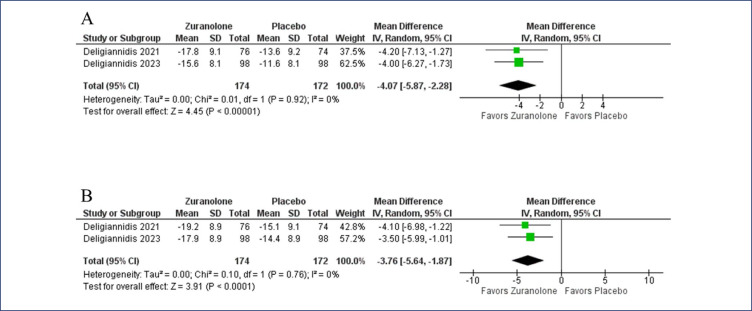
The delta in HAM-D score from baseline was significantly more favorable in the zuranolone group at both 15-days (A) and 45-days (B)

**Figure 4:**
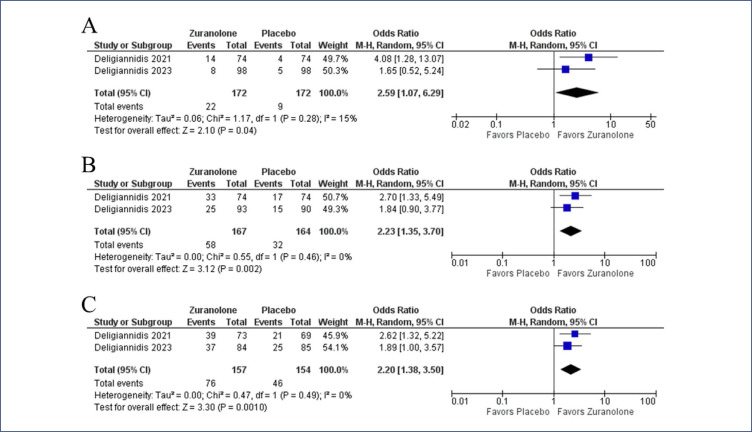
Remission of PPD symptoms was higher in the zuranolone group at 3 days (A), 15 days (B) and 45 days (C)

**Figure S1:**
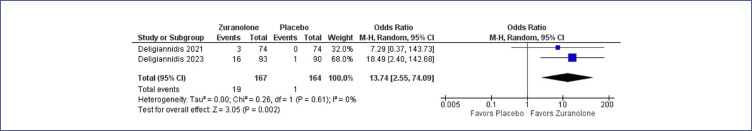
A reduction in the dose of antidepressant therapy was more common in the zuranolone group.

In the pooled analysis, zuranolone improved the CGI response rate (OR 2.31, 95% CI 1.49-3.58; p< 0.001; I^2^= 0%, p= 0.98) (Figure 2). The delta in HAM-D score from baseline to 15-days favored the zuranolone group (MD −4.07, 95% CI −2.28 to 5.87; p<0.001; I^2^= 0%, p= 0.92). Similarly, the difference from baseline to 45-days was also improved in the zuranolone group (MD −3.76, 95% CI −5.64 to −1.87, p<0.001; I^2^= 0%, p= 0.76) (Figure 3). Remission in depressive symptoms at 3 days (OR 2.59, 95% CI 1.07-6.29, p= 0.04; I^2^= 15%, p= 0.28), 15 days (OR 2.23, 95% CI 1.35-3.70, p= 0.002; I^2^= 0%, p= 0.46), and 45 days (OR 2.20, 95% CI 1.38-3.50, p= 0.001; I^2^= 0%, p= 0.49) (Figure 4) was significantly higher in the zuranolone group. A reduction in the dose of antidepressants was more frequent with zuranolone therapy (OR 13.74, 95% CI 2.55-74.09, p= 0.002; I^2^= 0%, p= 0.61) (Online Resource 1: Figure S1).

### Safety outcomes

**Figure S2:**
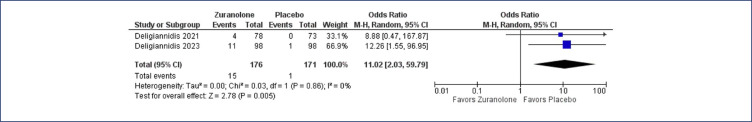
Sedation was significantly more common in the zuranolone group.

**Figure S3:**
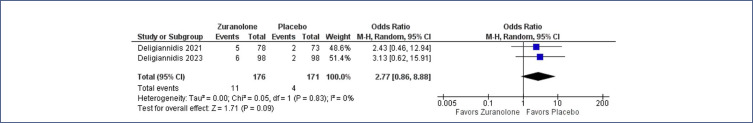
Forest plot of summary of crude ORs and 95% CIs for diarrhea for zuralonone *versus* placebo

**Figure S4:**
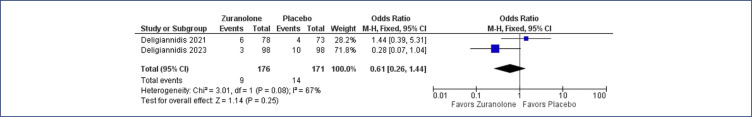
Forest plot of summary of crude ORs and 95% CIs for dizziness for zuralonone *versus* placebo

**Figure S5:**
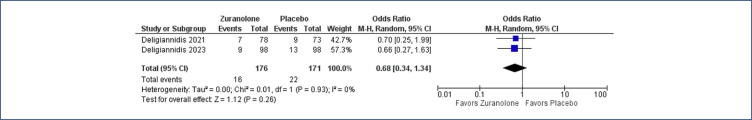
Forest plot of summary of crude ORs and 95% CIs for headache for zuralonone *versus* placebo

**Figure S6:**
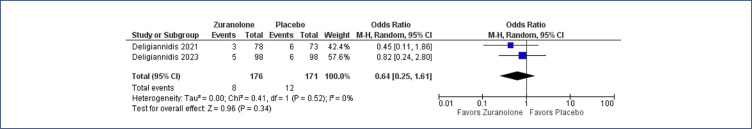
Forest plot of summary of crude ORs and 95% CIs for nausea for zuralonone *versus* placebo

**Figure S7:**
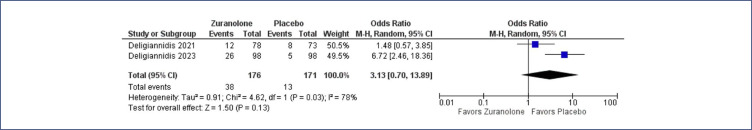
Forest plot of summary of crude ORs and 95% CIs for somnolence for zuralonone *versus* placebo

**Figure S8:**
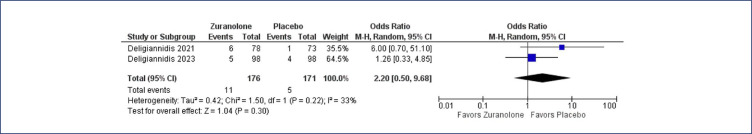
Forest plot of summary of crude ORs and 95% CIs for Upper respiratory tract infection for zuralonone *versus* placebo

Zuranolone increased the risk of sedation (OR 11.02, 95% CI 2.03-59.79, p= 0.005; I^2^= 0%, p= 0.86) (Online Resource 1: Figure S2). There were no significant differences between groups for the outcomes of diarrhea (OR 2.77, 95% CI 0.86-8.88, p= 0.09; I^2^= 0%, p= 0.83; Online Resource 1: Figure S3), dizziness (OR 0.61, 95% CI 0.26-1.44, p=0.25; I^2^= 67%, p= 0.08; Online Resource 1: Figure S4), headache (OR 0.68, 95% CI 0.34-1.34, p = 0.26; I^2^= 0%, p= 0.93; Online Resource 1: Figure S5), nausea (OR 0.64, 95% CI 0.25-1.61, p= 0.34; I^2^= 0%, p= 0.52; Online Resource 1: Figure S6), somnolence (OR 3.13, 95% CI 0.70-13.89, p= 0.13; I^2^= 78%, p= 0.03; Online Resource 1: Figure S7), and upper respiratory tract infections (OR 2.20, 95% CI 0.50-9.68; p= 0.3; I^2^= 33%, p= 0.22; Online Resource 1: Figure S8).

### Quality Assessment

**Figure S9:**
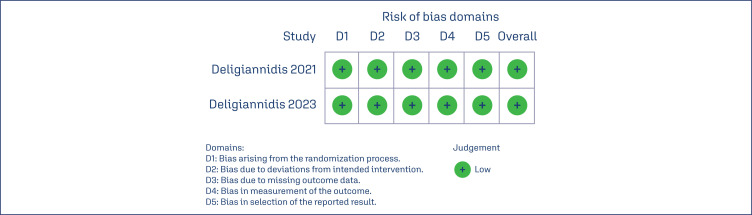
ROB-2

Both studies were classified as low risk of bias in all domains of the RoB2 tool, yielding an overall low risk of bias (Online Resource 1, Figure S9).

## Discussion

In this meta-analysis, we evaluated the safety and effectiveness of zuranolone in a cohort of 346 pregnant women diagnosed with major depression during the third trimester or up to 4 weeks post-delivery. We compared zuranolone to a placebo to assess maternal outcomes. Our primary findings indicate an improvement with zuranolone, relative to placebo, in (1) CGI response rates; (2) remission rates at 15- and 45-days, measured both as a continuous and as a binary endpoint; and (3) the reduction in doses of antidepressants.

The mechanisms of action of zuranolone include inhibitory GABAergic activity and excitation of glutamatergic neurons, which play an essential role in cortical neural activity.^(7)^ GABA neurons are found in areas associated with memory, emotions, motivation, pleasure, and decision-making. Neuroactive steroids (NAS) act on GABA neurons and even serotonin receptors, being able to regulate functions related to mood control and pain.^(11)^ Both GABA and NAS, when downregulated, are associated with psychiatric disorders, such as major and postpartum depression,^(12)^ and even neurodegenerative and inflammatory brain diseases, such as Alzheimer.^(13)^

A systematic review comprising 595 patients assessing the efficacy of selective serotonin uptake inhibitors for women with PPD found no benefit of fluoxetine, sertraline and paroxetine in symptoms, as measured by depression scales. Therefore, zuranolone may represent the first effective oral treatment for PPD.^(14)^ The only other FDA-approved treatment for severe postpartum depression is "brexanolone", another neuroactive steroid GABA receptor modulator. However, brexanolone is administered intravenously over a 60-hour period, which represents a significant challenge, especially for new mothers.^(6)^ Zuranolone has been mentioned in Brazilian government updates on the treatment of major depression,^(15)^ yet its arrival in Brazil is still awaited.

Zuranolone is generally well-tolerated.^(4,16)^ In our study, sedation was statistically more common in women treated with zuranolone. Importantly, the effect of zuranolone on sedation may be dose-dependent, and less frequent with a 30-mg dose, as compared with 50-mg.^(4,10)^ This dosage-dependent increase in side effects is supported by a meta-analysis of 1789 patients, which also demonstrated greater efficacy of zuranolone in PPD-related major depressive disorder compared to MDD in the general population.^(17)^ No effects on drop-out rates or in the incidence of side effects were observed with higher doses (50mg) in the general population with MDD.^(17)^ Further dosage impact needs to be assessed.

To the best of our knowledge, this is the first comprehensive meta-analysis of these trials evaluating all outcomes for zuranolone therapy for PPD. A prior meta-analysis of zuranolone for patients with major depression performed a subgroup analysis for those with PPD; however, the prior study did not evaluate key outcomes of interest, such as CGI response rate, delta in HAM-D score from baseline to 15-days and 45-days, remission in depressive symptoms at 3 days, 15 days and 45 days, dose reduction, and they didn't evaluate the side effects isolated (sedation, diarrhea, dizziness, headache, nausea, somnolence and upper respiratory tract infections).^(17)^

While Zuranolone showed efficacy in improving PPD symptoms compared to placebo, with indications of a manageable safety profile, the study's scope was limited to two U.S. trials with a short follow-up period of 45 days. Long-term studies and broader population analyses are needed. Additionally, the small number of studies precluded more extensive statistical analysis of population subgroups, such as the effect at different age groups and potential interaction of treatment with baseline severity of PPD.

## Conclusion

Our findings suggest that zuranolone might be associated with a higher rate of clinical remission in women with PPD as compared with placebo. This novel therapy could be a valuable option in the treatment of PPD.

## Appendix

**Chart S1:** Search strategies used for databases searched

Database	Search Strategy
PubMed, Embase, and Cochrane	(zuranolone OR "SAGE-217" OR 3alpha-Hydroxy-3beta-methyl-21-(4-cyano-1H-pyrazol-1’-yl)-19-nor-5beta-pregnan-20-one" OR "1-(3.alpha.-hydroxy-3.beta.-methyl-20-oxo-19-nor-5.beta.-pregnan-21-YL)-1H-pyrazole-4-carbonitrile" OR "1H-pyrazole-4-carbonitrile, 1-((3.alpha.,5.beta.)-3-hydroxy-3-methyl-20-oxo-19-norpregnan-21-yl)-" OR "3beta-Methyl-21-(4-cyano-1H-pyrazol-1’-yl)-19-norpregnanolone")
